# Comparative Exploratory Analysis of Intrinsically Disordered Protein Dynamics Using Machine Learning and Network Analytic Methods

**DOI:** 10.3389/fmolb.2019.00042

**Published:** 2019-06-12

**Authors:** Gianmarc Grazioli, Rachel W. Martin, Carter T. Butts

**Affiliations:** ^1^California Institute for Telecommunications and Information Technology (Calit2), University of California, Irvine, Irvine, CA, United States; ^2^Department of Chemistry, University of California, Irvine, Irvine, CA, United States; ^3^Department of Molecular Biology and Biochemistry, University of California, Irvine, Irvine, CA, United States; ^4^Department of Computer Science, University of California, Irvine, Irvine, CA, United States; ^5^Department of Sociology, Statistics, and Electrical Engineering and Computer Science, University of California, Irvine, Irvine, CA, United States

**Keywords:** machine learning, intrinsically disordered proteins, molecular dynamics, amyloid fibrils, amyloid beta, protein structure networks, support vector machines, clustering

## Abstract

Simulations of intrinsically disordered proteins (IDPs) pose numerous challenges to comparative analysis, prominently including highly dynamic conformational states and a lack of well-defined secondary structure. Machine learning (ML) algorithms are especially effective at discriminating among high-dimensional inputs whose differences are extremely subtle, making them well suited to the study of IDPs. In this work, we apply various ML techniques, including support vector machines (SVM) and clustering, as well as related methods such as principal component analysis (PCA) and protein structure network (PSN) analysis, to the problem of uncovering differences between configurational data from molecular dynamics simulations of two variants of the same IDP. We examine molecular dynamics (MD) trajectories of wild-type amyloid beta (Aβ_1−40_) and its “Arctic” variant (E22G), systems that play a central role in the etiology of Alzheimer's disease. Our analyses demonstrate ways in which ML and related approaches can be used to elucidate subtle differences between these proteins, including transient structure that is poorly captured by conventional metrics.

## 1. Introduction

Molecular dynamics (MD) simulations, either alone or guided by experimental data, have greatly enhanced our ability to probe molecular motions at the atomic scale. Unfortunately, these advances can also lead to the creation of a map that is almost as complex as the territory it describes: as simulation methodology has improved, the need for approaches to analyze and make sense of increasingly information-rich simulated trajectories has grown. This is particularly true in the case of intrinsically disordered proteins (IDPs), where recent developments in the combined use of simulation methods with NMR (Dedmon et al., 2005; Salmon et al., 2010; Salvi et al., 2016) and small angle x-ray scattering data (Sibille and Bernadó, 2012) have led to a proliferation of configurational information. The dynamics of and transient conformations explored by IDPs are often extremely high dimensional and are not always well described by the standard vocabulary of structural biology. Machine learning and network analytic approaches offer potentially valuable ways of addressing such problems by facilitating (respectively) the detection of systematic patterns in high-dimensional data and the representation and modeling of complex structures that do not follow simple, regular motifs (e.g., alpha helices or beta strands). In this paper, we show how tools drawn from both traditions can give purchase on the comparative exploratory analysis of molecular dynamics trajectories from protein variants, yielding insights that would be difficult to obtain using more conventional methods. We illustrate our approach using simulations of the wild type (WT) Aβ_1−40_, a well-known intrinsically disordered protein and its E22G (“Arctic”) variant, which is implicated in familial Alzheimer's disease (Nilsberth et al., 2001), and which has been a system of interest for many previous molecular dynamics studies (Cecchini et al., 2006; Lam et al., 2008; Urbanc et al., 2010).

The majority of proteins have a well-defined structure-function relationship, whereby the protein's biological role is contingent on it being correctly *folded* into its flexible, but locally stable, functional configuration. By contrast, intrinsically disordered proteins (along with proteins possessing a significantly large intrinsically disordered region) owe their function to not being confined to a small number of stable regions of configuration space. For example, many signaling proteins are able to bind a wide variety of targets due to their intrinsic disorder (Iakoucheva et al., 2002). The study of IDPs presents challenges inherent to both the molecular systems themselves and the standard conventions used by the scientists who study proteins. In addition to the difficulty of distilling down the complex motions of these “moving targets” of structural biology to some intuitable form, there are additional difficulties due to the standard descriptive and experimental toolkits used by structural biologists and chemists, from Ramachandran plots to X-ray crystallography, being tailored toward gaining insight about proteins within the paradigm of a small number of favored static configurations. Thus, if we wish to search for latent order characteristics of a particular IDP, we must establish methodologies for characterizing and interpreting IDP data. Such problems, where vast amounts of high-dimensional unstructured data is available for a set of known classes (e.g., WT class vs. E22G class) are the exact situations where machine learning algorithms excel. In fact, a great deal of progress has been made in the development of ML-based technologies for the interpretation of chemical and biochemical systems, such as automated optimal partitioning of configuration space for building kinetic models (Grazioli et al., 2017), clustering-based methods for building Markov models of protein folding (Husic and Pande, 2017), protein conformational space mapping with self-organizing maps (Bouvier et al., 2014), protein-ligand interaction scoring (Ragoza et al., 2017), automating the definition of atom types in molecular mechanics force fields (Zanette et al., 2018), and even the *inverse design* of materials, using ML to guide material design, given a set of desired material properties (Sanchez-Lengeling and Aspuru-Guzik, 2018).

A related problem is summarizing the transient structures of IDPs in a way that is reductive enough to provide useful simplification while still being flexible enough to accommodate a wide range of irregular structural configurations. Network representations, which have been extensively studied in the context of human social networks (Wasserman and Faust, 1994), provide a natural tool for this purpose. Most relevant to IDP behavior are protein structure networks (PSNs), which represent protein structures in terms of relationships (e.g., bonded or non-bonded interactions) among groups of atoms (e.g., moieties, residues, or whole secondary structure elements). PSNs are useful for coarse-graining protein structure while retaining topological information describing internal contacts, and have been employed to rapidly identify enzymes with distinct but non-obvious structural features (Butts et al., 2016), characterize local packing characteristics distinguishing closely related enzyme classes (Unhelkar et al., 2017), distinguish structural features particular to thermophilic vs. mesophilic proteins (Brinda and Vishveshwara, 2005), analyze simulation trajectories (Benson and Daggett, 2012), and predict differences in overall protein (Atilgan et al., 2001; Jacobs et al., 2001) and active site (Duong et al., 2018) flexibility, among other tasks (Csermely et al., 2012). PSNs can be modeled using statistical techniques adapted from social network analysis (Yaveroğlu et al., 2015), allowing for very flexible and computationally efficient identification of structural biases distinguishing groups of proteins, tests of hypotheses relating to protein topology, and simulation of PSN structure. Here we leverage these techniques to uncover differences in the respective energy landscapes of Aβ_1−40_ wild type and E22G.

In addition to providing broadly applicable methodology, we also present applications of this approach to the elucidation of the dynamic, and often subtle, characteristics of wild-type Aβ_1−40_ and its variant E22G that lead to their distinct behavior in solution, despite their being identical in all but one amino acid. Although the present discussion is focused on applying our methodologies to IDPs, it is noteworthy that there are also examples of well-folded proteins, like TEM-1 β-lactamase (Roccatano et al., 2005) or ZASP PDZ (Fratev et al., 2014), where the structural changes caused by point mutations can also be very difficult to discern in molecular simulations, despite the mutations having known physiological effects. Thus, the approaches discussed here may have applicability beyond the IDP case. The remainder of the paper is organized as follows: we begin by applying simple and well-established methods for comparing data generated by molecular dynamics simulations of both WT Aβ_1−40_ and the E22G variant (e.g., Ramachandran plots), highlighting their limitations in the context of intrinsically disordered proteins. Although the two proteins seem at first blush to exhibit nearly identical behavior, we show how support vector machines (SVMs) can be employed to construct a metric that readily distinguishes them. Projection of conformations obtained from structures of Aβ fibrils onto this metric can then be used to predict differences in fibrillization behavior. Moving from torsion angles to topology, we employ exponential family random graph models (ERGMs) to characterize the properties of favorable transient structures in Aβ_1−40_ residue-level PSNs, and use this to explore the structures most energetically favored by WT vs. E22G (and vice versa). We then close with a demonstration of how joint *k*-means clustering of conformations from long WT and E22G trajectories and network analysis of the Markov transition graph on the resulting conformational states reveals substantial differences in dynamics that are not apparent on casual inspection. Additional technical details regarding our simulations and analysis are provided in the following section, and we conclude with a discussion of our findings and how approaches such as these can be used to select targets for further experimental biophysical characterization and structural biology.

## 2. Results

### 2.1. Exploring the Torsion Angle Space of Energy Minima

**Figure 1:**
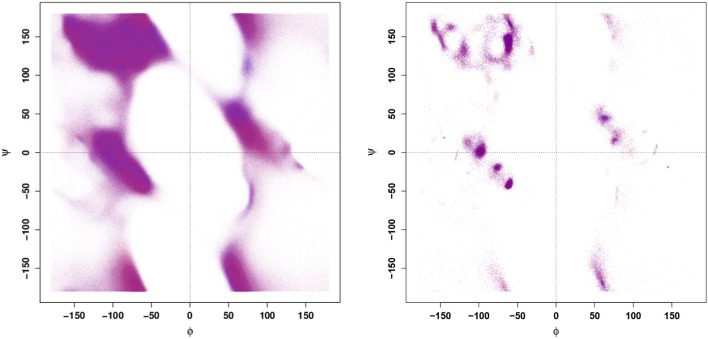
Ramachandran plots for all conformations **(Left panel)** and conformations of local minima **(Right panel)**. Points are colored by variant (blue - WT, red - E22G); apparent purple color indicates near identical distribution of torsion angles.

Prior to applying more complex, ML-based techniques for identifying the characteristic differences between the configurational dynamics of the WT and E22G variants, it is reasonable to first apply more established approaches toward that same end. Thus, we begin by calculating a Ramachandran plot (Figure 1) from a large set of configurations generated by MD simulations from a highly dispersed set of seed conformations (details provided in section 4), as well as from conformations associated with large samples of local energy minima. It is clear from the data shown in Figure 1 that WT and E22G cannot be distinguished by their distributions in Ramachandran space. This result illustrates the core problem of exploratory analysis of intrinsically disordered proteins: many of the simple and familiar tools of structural biology exploit the fact that folding confines typical proteins to a narrow range of conformations, and the lack of such confinement leaves them with little signal to leverage.

**Figure 2:**
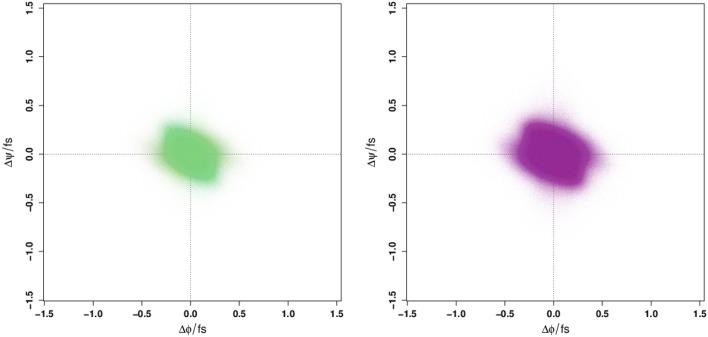
Angular velocity distributions by dihedral angle colored by residue index **(Left panel)** and by protein **(Right panel)**. No systematic variation is visible in either case.

Given that the Ramachandran plot does not display any obvious differences that could be used to distinguish between WT and E22G conformations in torsion angle space, it is natural to ask whether these variants might still be distinguished by the distribution of their *angular velocities* in the same space. Employing a large number of trajectories initialized from a set of widely dispersed local minima (see section 4), we plot the distribution of local ψ and ϕ angular velocities in the equivalent of a Ramachandran space (Figure 2). As can be seen, the resulting velocity distribution is homogeneous both by residue index (left) and by variant (right), with the points colored for each attribute overlapping so completely that they appear to form a single undifferentiated distribution. Plainly, this property cannot differentiate between WT and E22G. Moreover, the similarity in velocity distributions between variants suggests that differences in the energy landscape associated with the E22G mutation are extremely subtle, despite its known differences in aggregation behavior relative to wild type (Lord et al., 2006; Norlin et al., 2012).

The lack of distinguishing features in either the Ramachandran space of conformations or the “differentiated Ramachandran” space of angular velocities highlights the subtle nature of differences in IDP behavior, and points to the need for more flexible—and high dimensional—techniques to identify differences. We now turn to a family of kernel learning methods that are well-suited to this purpose.

### 2.2. Finding Relatively Favored Conformations via SVM

The observation that WT and E22G Aβ_1−40_ differ by a single residue, yet exhibit differing propensities for fibrillization in experiments (Norlin et al., 2012), seems to imply that the conformations they sample in solution must originate from differing equilibrium distributions in configuration space. Further, we note that if a configuration is defined as a vector of all torsion angles for residues 1 through 40, the respective distributions for WT and E22G both “live” in the same coordinate space. Thus, we may posit some *characteristic axis*, onto which any configuration in the shared torsion angle space can be projected, where points at one extreme are most characteristic of WT (and least likely to be sampled by E22G) and points on the other extreme are most characteristic of E22G (and least likely to be sampled by WT). If we, for the sake of argument, were to imagine that the sets of conformations sampled by each variant were linearly separable—i.e., a separating hyperplane in torsion angle space could be placed between them with all WT points on one side and all E22G points on the other—such an axis would be trivial to define: it would be the vector normal to the separating hyperplane. Unfortunately, the condition of linear separability is an unrealistic assumption for two systems that are both highly similar and high dimensional, and the Ramachandran analysis of Figure 1 suggests that it is inapplicable here. However, we could consider an alternative version of our construction, in which we nonlinearly map our torsion angle space into an alternative space (called a *feature space*) in which our conformations *are* linearly separable and then find the characteristic axis within this modified space. The resulting characteristic axis would no longer take a simple form in our original space (the *input space*), but we could nevertheless use it to “score” hypothetical conformations for similarity to WT vs. E22G by mapping them into the feature space and finding their projection onto the characteristic axis in that space.

Finding transformations of this type in high-dimensional data is a central problem of *kernel learning* (Scholkopf et al., 1999), and identifying a “characteristic axis” like the one envisioned above is a natural application of *support vector machines* (SVMs) (Vapnik, 2013). In a classification context, SVMs seek maximum-margin separating hyperplanes between sets of observations, with the characteristic axis corresponding to a quantity (often called the *decision value*) that is used to predict class membership. While “pure” SVMs are linear algorithms, kernelized SVMs (i.e., SVMs operating on kernel-transformed inputs) are powerful tools for finding complex separating surfaces (or, in the case of imperfect separability, approximate separating surfaces) in more general contexts.

**Figure 3:**
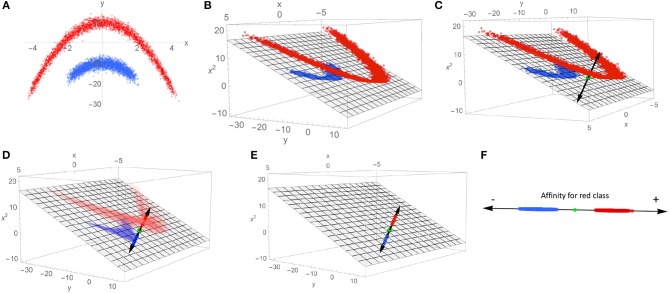
This schematic demonstrates the process of mapping linearly non-separable data to a single coordinate that indicates membership to each class. **(A)** An example of linearly non-separable 2D data with two classes indicated by red and blue dots. **(B)** An exact mapping of the points shown in **(A)**. To a 3D feature space, where the data is linearly separable by the plane shown as gray mesh. **(C)** A vector normal to the separating plane (black double-headed arrow) is introduced, along with the intersection of the separating plane and normal vector (green dot). **(D)** The points in the 3D feature space are projected onto the normal vector. **(E)** The projected points on the characteristic axis are shown in the 3D space. **(F)** The characteristic axis now serves as the reference frame for a scalar scoring indicating a point's affinity for one class vs. the other.

A heuristic illustration of how SVMs can be used to extract a characteristic axis from linearly non-separable data classes is shown in Figure 3, as an aid to intuition. Note that in the input space {*x, y*} (Figure 3A), no single plane can be defined that perfectly separates the blue class from the red class. By mapping the data to the higher-dimensional space of all polynomials in *x* and *y* (truncated to the subspace {*x, y, x*^2^} in Figure 3B, chosen for visualization purposes), this same data set is now linearly separable. Such a mapping onto quadratic functions of the inputs constitutes a polynomial kernel of order 2, with mapping into higher-order polynomials corresponding to higher-order kernels; mapping to polynomial functions of arbitrary order can be performed by selection of e.g., the Gaussian or radial basis function (RBF) kernel, whose basis set can be interpreted in terms of Taylor series expansions of exponential functions. Such an expansion can in principle find a separating hyperplane for any point set (subject to regularity conditions), making the RBF kernel a so-called “universal” kernel. With a separating plane now defined in the kernel-transformed feature space, the data points can be projected onto the vector normal to that plane (C). This vector is our characteristic axis, with the 0 point corresponding to the point of maximum margin when dividing the two classes.

To apply this idea to the case of our Aβ variants, we trained an SVM classifier under a RBF kernel to distinguish low-energy conformations of WT (obtained by independent annealing trajectories seeded with an overdispersed sample of conformations obtained via a high-temperature trajectory) from those of E22G (see section 4 for details). To gain insight into conformations that are relatively favorable for E22G vs. WT, we approximately linearize the decision surface (i.e., the pre-image of the separating hyperplane in the input space) and examine its characteristics averaged over the E22G/WT conformations that are closest to it. Specifically, we identify the *support vectors* from the SVM solution (i.e., the data cases with non-zero weight, from which the decision surface is defined), and identify pairs of WT and E22G support vectors that are as close as possible within the input space (as measured by Euclidean distance between inputs). Each of these pairs can be envisioned as straddling the decision surface, with no other pair being strictly closer to it (since, if so, at least one point in the pair would not be a support vector). Taking the difference of properties between one conformation in the pair and the other thus allows us to approximate the gradient of the decision surface with respect to those properties in the original (input) space, at some point between the conformation pair. Considering the distribution of such differences over all such pairs then gives us insight into the properties that typically do (or do not) typically distinguish E22G trajectories from those of WT.

**Figure 4:**
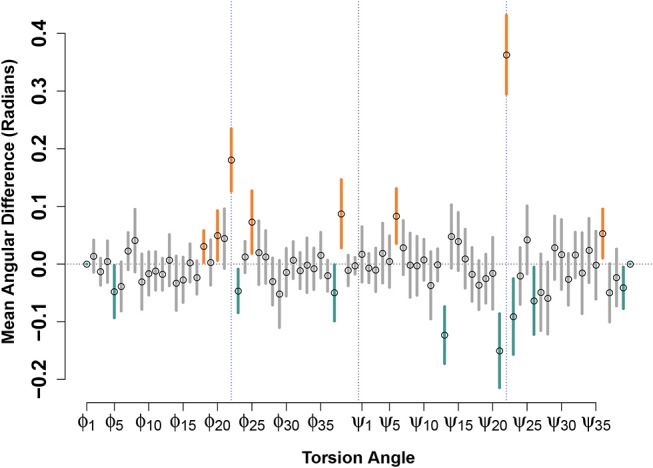
Mean angular difference in torsion angles between WT and E22G minima, across the SVM decision boundary. For each torsion angle, *y* axis values show the average across all support vector pairs of the angular difference in WT and E22G values; 95% confidence intervals indicated by thick lines (orange values significantly positive, teal values significantly negative). Prominent deviations are seen at the mutation site (blue dotted lines) but can also be seen at many other locations along the backbone.

Figure 4 shows the result of such a calculation performed for the (circular) mean differences in torsion angles between paired E22G and WT support vectors, for the low-energy conformation model. Although many angles show no significant differences—indicating that, on average, there is no *net* contribution of position on this angle to relative favorability—some show a clear and systematic difference across the decision boundary. Perhaps most notable are the torsion angles for ϕ_22_ and ψ_22_, both of which show positive change when moving across the decision boundary from the WT to the E22G side. (Put another way, ψ_22_ tends to be turned approximately 0.35 radians to the right within E22G minima from its value in similar WT trajectories). In addition to confirming the intuition that the substantial loss of side chain steric hindrance brought about by the E22G mutation alters the local backbone curvature at the mutation site, our analysis allows us to focus on the torsion angle changes that best distinguish otherwise similar local minima. For instance, we also see significant increases in ϕ angles for residues 18, 20, 25, and 38, and decreases for residues 5, 23, and 37, showing systematic effects on several (but not all) sites along the backbone. Similarly, we see significant additional increases in ψ angles for residues 6 and 36, and decreases for residues 13, 21, 23, 26, and 39, showing that the two torsion angles are affected differently by the mutation but that those effects show signs of clustering (e.g., the relatively numerous angular differences near the mutation site, or for residues 37-39 at the C terminus).

**Figure 5:**
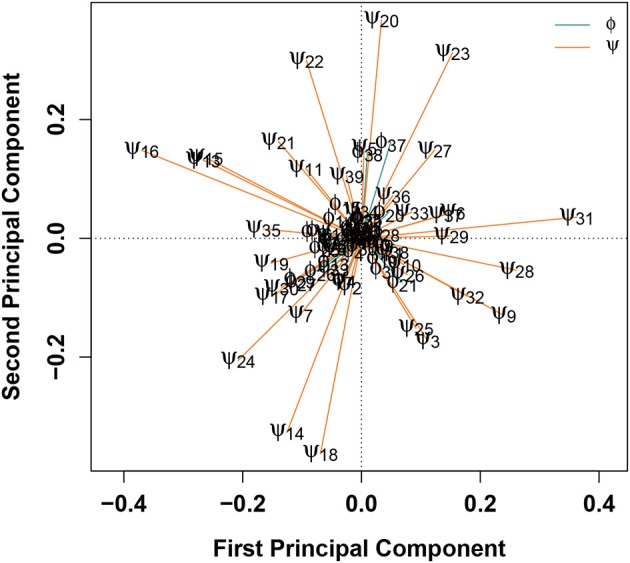
PCA of angular differences in torsion angles between WT and E22G minima, across the SVM decision boundary. Vectors show ϕ (teal) and ϕ (orange) torsion angle loadings on the first two principal components; angles with similar loadings tend to show similar patterns of differences for E22G vs. WT conformations.

Another method for determining which degrees of freedom contribute most substantially to the classification of a configuration as belonging to either WT or E22G is to combine SVMs with principal component analysis (PCA), as shown in Figure 5. In this treatment, the differences in torsion angles between WT and E22G minima across the SVM decision boundary are processed using PCA, resulting in a new reference frame in which the principal components are linear combinations of the original dimensions that begin with the direction of maximum variance and proceed in subsequent orthogonal directions in order of diminishing variance (Pearson, 1901). Thus, plots of the first two principal components, such as Figure 5, display the two directions through the space of torsion angle differences that best summarize (in a least squares sense) the total pattern of variation in torsion angle differences across the decision surface. The loadings on these components hence provide information on which angles contribute most to these directions, and on the sense of that contribution (i.e., positive or negative).

Figure 5 shows that, with the exceptions of ϕ_37−38_, the first two components are strongly dominated by the ψ torsion angles. This result is consistent with the greater variance in ψ relative to ϕ in standard protein secondary structures, but it was not observable from the Ramachandran plot of Aβ conformations given in Figure 1. The strongest contrasts seen are between: ψ_13_ and ψ_15−16_ (left) and a group of angles including ψ_31−32_, ψ_3_, ψ_9_, ψ_25_, and ψ_28_ (right); and ψ_20_ and ψ_22−23_ (top) and ψ_14_, ψ_18_, and ψ_24_ (bottom). The first contrast involves a cluster of residues marking the N-terminal end of a stretch of residues forming a (transient) α-helix in a solution-state NMR structure (PDBID: 2LFM) (Vivekanandan et al., 2011) vs. a collection of several residues in the terminal regions of the protein. The second contrast, interestingly, pits a cluster of residues at the C-terminal end of the aforementioned helix-forming region with three residues spanning it (two at either end and one in the middle). This suggests one mode involving the extent of helical structure in range of residues 14-23, and another involving a broader pattern of curvature throughout the protein. By identifying such patterns, we can potentially focus attention on particular conformational features that are differentially favored by E22G vs. wild-type Aβ.

One obvious application for a score distinguishing WT and variant conformations is in screening for the potential to exhibit distinct patterns of fibrillization. Fibrillization is difficult to probe directly via MD trajectories, due to the long timescales and large atom counts involved, and fibrillization experiments with new systems are costly. In particular, structure determination efforts are time-consuming and often require technological innovations to achieve. Although amyloid fibrils by definition form a common cross-β structure, they often differ in detailed structural topology. Therefore, given a new variant with potential clinical significance, it is useful to be able to obtain some indication of whether or not it is likely to form fibrils with the same structural topology as the wild-type protein. While the SVM analysis conducted here cannot provide a definitive answer to this question, it can tell us (based on the sets of trajectories available) whether known fibril structures involve monomeric conformations that are *more characteristic of wild-type than the variant*. If WT and the variant (here E22G) have similar affinity for a particular set of fibrillar conformations, then this suggests that the variant will have a similar propensity to produce such fibrils in practice; however, if the affinity differs strongly between WT and the variant, then this may indicate a difference in the propensity to produce fibrils of this topology.

**Figure 6:**
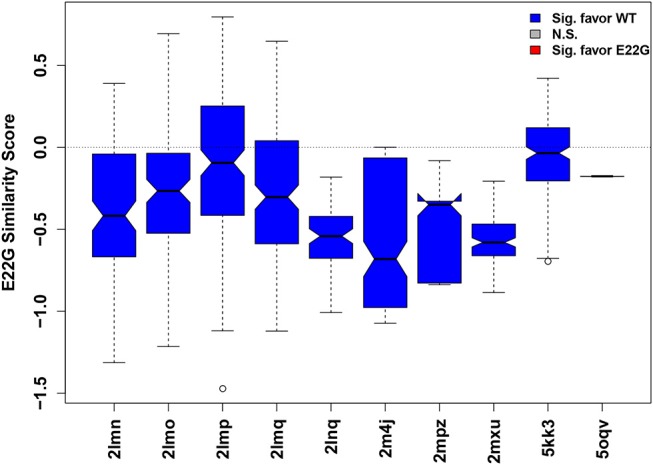
Distribution of similarity scores for conformations from known fibril structures (Aβ residues 15–40). Higher values (above dotted mid-line) indicate greater net E22G affinity, while lower values indicate greater net WT affinity. The overwhelming majority of conformations are more characteristic of WT minima than E22G minima, suggesting that there is a different fibrillization pattern for E22G, making this variant a high priority for future structural studies.

Such an approach is illustrated in Figure 6, where the relative similarity of fibrillar conformations to E22G vs. WT (as expressed by projection onto the characteristic axis) is shown for all conformations from 10 Aβ fibril structures found in the Protein Data Bank. While some individual configurations appear more favorable for E22G than WT (positive values), all fibril structures were overall significantly more typical of WT solution minima than the minima observed for E22G (hence all plot markers are blue in Figure 6), suggesting that the latter has a different fibrillization pattern. Interestingly, the two non-wild type fibrils included (PDBIDs 2LNQ and 2MPZ, both of the D23N or “Iowa” variant) show particularly strong relative affinity for WT vs. E22G, suggesting that E22G's fibrillization behavior differs from that of both variants. These results are compatible with experimental findings that have previously suggested that E22G may have a different fibrillization mode from WT, potentially proceeding through a different oligomeric precursor. A study employing a variety of biophysical techniques concluded that aggregation of this species proceeds via a characteristic type of on-pathway intermediates and then quickly progresses to a highly polymorphic variety of fibrils (Norlin et al., 2012), making high-resolution structure determination difficult. Given the time and expense necessary for solving atomic-resolution structures of even a single fibrillar conformation, measures of potential dissimilarity in fibrillization behavior are useful tools for choosing new structural targets. Disease-relevant variants, such as E22G, that are likely to occupy one or more novel fibril topologies can be considered high-priority targets for further structure determination efforts. It is important to reiterate here that the similarity scores for each fibril type represent how similar each fibril structure is to WT vs. E22G, thus two fibril structures whose similarity scores are close in value may or may not be similar to each other.

As with the decision surface, we can gain some additional insights regarding the local factors that tend to lead fibrillar conformations to be more favorable for E22G vs. WT by local linearization. In this case, we proceed by regressing the similarity score (projection onto the characteristic axis) for each fibrillar conformation onto the input features of each conformation (the real and imaginary components of its torsion angles). The coefficients from this regression represent the mean gradient of the score over the fibrillar conformations; to convert these into statements involving the original torsion angles, we express the gradient elements associated with each angle (i.e., ${\hat{\beta }}_{i}\sin\left(\theta_{i}\right)+{\hat{\beta }}_{i}^{\prime }\cos\left(\theta_{i}\right)$, for angle θ_*i*_ with regression coefficients ${\hat{\beta }}_{i},{\hat{\beta }}_{i}^{\prime }$) in the periodic form *b*_*i*_ sin(θ_*i*_−*y*_*i*_) [where $b_{i}=\sqrt{{\hat{\beta_{i}}}^{2}+{\hat{\beta_{i}^{\prime }}}^{2}}$ and $y_{i}=\tan^{-1}\left({\hat{\beta }}_{i}^{\prime }/{\hat{\beta }}_{i}\right)$]. Intuitively, the modulus *b*_*i*_ scales the absolute magnitude of the contribution of local changes to the *i*th torsion angle to changes in the expected similarity score, while the argument *y*_*i*_ defines a *reference angle* or *angular offset* such that small increments above *y*_*i*_ increase similarity to E22G, while small decrements below *y*_*i*_ decrease it.

**Figure 7:**
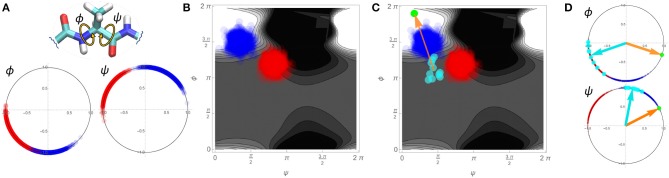
Schematic demonstration of the linearized similarity score used in Figure 8 using a simplified 2-dimensional example. **(A)** 1000 sampled conformations for two protein variants (blue and red dots) on two torsion angles. **(B)** Depiction of blue and red conformations in Ramachandran space, with an SVM-derived affinity score (background gradient). Lighter regions are more typical of the blue variant, while darker regions are more typical of the red variant. **(C)** Mapping a set of hypothetical fibrillar conformations into Ramachandran space (cyan) allows them to be associated with affinity scores. Regression of affinity scores onto the underlying angular components yields the mean gradient of the affinity score local to the fibrillar conformations (orange arrow), which can be re-expressed in terms of a reference angle (green dot). **(D)** Re-expression of conformations in angular space, with reference angles (orange/green) and mean fibrillar conformations (cyan arrows) indicated. Direction of rotation relative to the reference indicates whether local angular changes (from the mean fibrillar conformation) would make the structure more “blue-like” vs. “red-like”.

A schematic detailing how such an approach is implemented is shown in Figure 7 using a single pair of ϕ and ψ torsion angles in a simplified, two-dimensional example. We consider two variants of a hypothetical protein (designated “blue” and “red”) with two torsion angles of interest, ϕ and ψ. The blue and red dots on the angular plots for ϕ and ψ in Figure 7A represent the values for these angles for 1,000 different configurations sampled for each variant. From these conformations we may create an affinity score surface by training an SVM classifier to classify blue vs. red configurations using the real and imaginary components of both angles ϕ and ψ as the training data ({*Re*(ψ), *Im*(ψ), *Re*(ϕ), *Im*(ϕ)}). Figure 7B shows this affinity score surface in ϕ, ψ space (lighter values favor blue, while darker values favor red), together with the sampled red and blue configurations from panel Figure 7A. Now, consider a set of comparable torsion angles obtained from fibril structures; these may also be projected into our angular space, as shown in Figure 7C (cyan points). Each fibrillar conformation can be assigned an affinity score based on its location on the affinity score surface, indicating the extent to which it is more typical of the blue vs. the red variant. Regressing the affinity scores of the fibrillar conformations on the underlying torsion angles yields the mean gradient of the affinity score surface in angular space across the fibrillar conformations (orange arrow). From this we can equivalently construct a set of reference angles (green dot) that expresses the torsion angles that would provide the average greatest tendency to be more blue-like (vs. red-like) in the vicinity of the fibrillar conformations. Returning to an angular representation, Figure 7D shows both the mean vectors for the fibrillar conformations (cyan) and the reference angles (orange/green) in polar space. Local rotations toward the reference angle are here associated with increasing “blueness,” while rotations away are associated with increasing “redness.”

**Figure 8:**
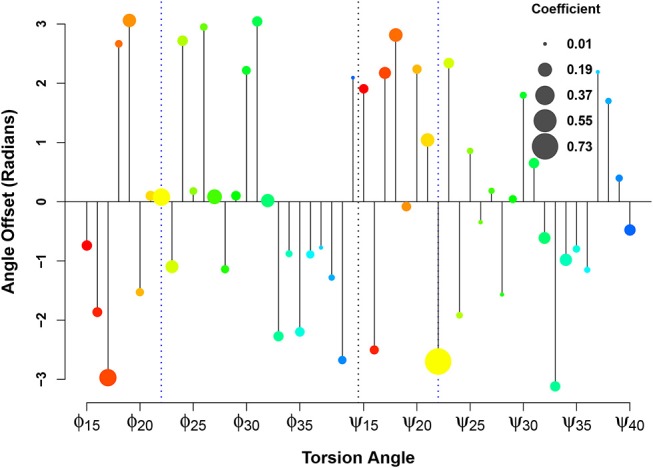
Linearization of the similarity score for fibrillar conformations. Relative affinity for E22G (vs. WT) increases as *b* sin(θ − *y*), where *y* is the angle offset (*y* axis), *b* is the weight coefficient (circle size), and θ is the torsion angle. The color spectrum from red to blue indicates the sequence position from N-terminal to C-terminal.

In applying this methodology at scale to the Aβsystem, we display these regression coefficients in the form of what we call an *orrery plot* in Figure 8. Each *y* axis value in the orrery plot gives the reference point for the associated torsion angle, while moduli are shown by point radius. Higher moduli indicate greater local contributions to the affinity score. (Note that, due to unreported residues in the fibrillar PDB structures, we limit our examination to residues 15-40). At a glance, the orrery plot tells us that the dominant local contributors to E22G similarity are the torsion angles at the mutation site, as well as angles such as ϕ_17_, ϕ_27_, ϕ_32_, ψ_18_, ψ_21_, and ψ_34_. The offset values show that not all torsion angles of the same type are in phase with each other (in the sense of having a common reference such that values higher or lower than the reference have the same impact on the similarity score), although some sets of residues do have very similar offsets. This may suggest particular groups of residues whose local conformations play a similar role in initiating or stabilizing fibril structure in wild-type Aβ. We also see many residues whose conformations do not seem to be strongly associated with relative affinity for wild-type vs. E22G (e.g., ϕ_37_ or ψ_24_), which suggests that differences in fibrillization behavior between the two variants are not likely to depend on the local conformations of these residues. The orrery plot thus provides us with guidance on the angular degrees of freedom that are more or less likely to distinguish protein variants with respect to their propensity to adopt fibrillar conformations.

### 2.3. Identifying Differences in Transient Structure via Network Analysis

As noted, a central challenge in the analysis of IDPs is their lack of the characteristic secondary structure motifs that are the primary point of reference for describing and comparing the tertiary structures of folded proteins. Although IDPs by definition lack stable secondary structure, they nevertheless form other types of transient structures that can be characterized. Transient structural features have been observed in weakly structured proteins (Williamson and Miranker, 2007; Lee et al., 2014) or partially folded intermediates (Teilum et al., 2002; Bernard et al., 2005), often using the sensitivity of NMR chemical shifts to local backbone conformation (Spera and Bax, 1991); such features are often found to resemble more stable structural elements formed upon interaction with a binding partner (Song et al., 2008). A natural approach to characterizing transient structural elements is via the use of residue-level PSNs to characterize the pattern of interactions among residues within sampled conformations, giving rise to coarse-grained representations that are flexible enough to represent the wide range of conformational variation exhibited by IDPs. A residue-level PSN is a network structure (or, more formally, a *graph*) whose nodes or vertices correspond to individual residues, and whose edges correspond to inter-residue contacts. Here, we define two residues *v*_*i*_, *v*_*j*_ to be in contact (*adjacent*) if there exists an atom *a*_*i*_ in residue *v*_*i*_ and atom *a*_*j*_ in residue *v*_*j*_ such that the inter-atomic distance between *a*_*i*_ and *a*_*j*_ is less than 1.2 times the sum of their respective van der Waals radii. We compute a PSN for each conformation in our set of respective WT and E22G energy minima, giving us an ensemble of PSNs (each a 40-node network) for each Aβ_1−40_ variant.

#### 2.3.1. Where Is Transient Structure Formed in E22G and WT?

A natural first question to address is where transient structure is potentially formed in the wild-type and variant proteins. While there are many types of local network structure that might be considered, we follow (Unhelkar et al., 2017) in using the degree *k*-cores of the PSN to indicate areas of cohesive interaction among residues. A (degree) *k-core* (Wasserman and Faust, 1994) is a maximum set of nodes such that every member of the set is adjacent to at least *k* other members of the set; the highest *k* such that vertex *v* belonging to the *k*th core of a graph is referred to as *v*'s *core number*, and is an indication of *v*'s embeddedness in locally cohesive structure. While *k*-cores need not be globally cohesive, high-numbered *k*-cores are composed of locally cohesive elements, and hence vertices with high core numbers represent residues belonging to regions of the protein connected by multiple redundant contacts. By contrast, vertices with low core numbers represent residues residing in regions that are at best very loosely connected.

**Figure 9:**
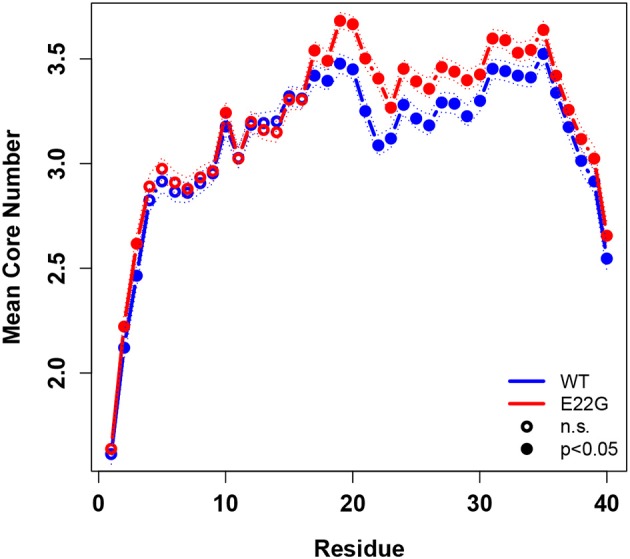
Mean degree core number by residue, WT vs. E22G, with 95% confidence intervals. Significant differences in core numbers are indicated by dark circles. E22G shows a region of markedly higher average cohesion involving internal residues in the C-terminal portion of the protein, suggesting a major role for these residues in the formation of transient structural elements that may be involved in aggregate formation.

To summarize global tendencies toward structure formation in the two variants, Figure 9 shows the mean core numbers for each WT and E22G residue, averaged over all minimum energy conformations in each respective set. Observed mean core numbers range from just over 1 at the N-terminus to over 3 in the internal region of the protein, falling again near the C-terminus. The relatively low core numbers near the termini are reflective of the high flexibility of these regions, though we observe a substantial and significant difference between the N-terminal and C-terminal regions (with the former being far less structured, on average, than the latter). In general, WT and E22G show very similar patterns of core structure throughout the N-terminal region, although E22G shows significantly higher core numbers for the majority of residues. The largest differences in core numbers are observed for a band of residues extending roughly from G15 to M35. Within this region, E22G produces substantially more local cohesion, on average, than WT. The elevated level of structure within this band for both variants may stem in part from interactions among the numerous nonpolar residues located within it, but the cross-variant difference points to a major role for E22 in destabilizing possibly aggregation-inducing local interactions throughout the C-terminal region. Although comparative experimental results are not available for these proteins, this central region of higher connectivity is consistent with the observations of Rosenman et al. (2013) from NMR experiments on the wild-type protein at low temperature. Based on measured J-couplings and molecular dynamics simulations, several frequently populated structural elements were observed, including a transient salt bridge between E22 and K28 [also observed by Rosenman et al. (2013)], which was observed in the minima of our wild-type models.

**Figure 10:**
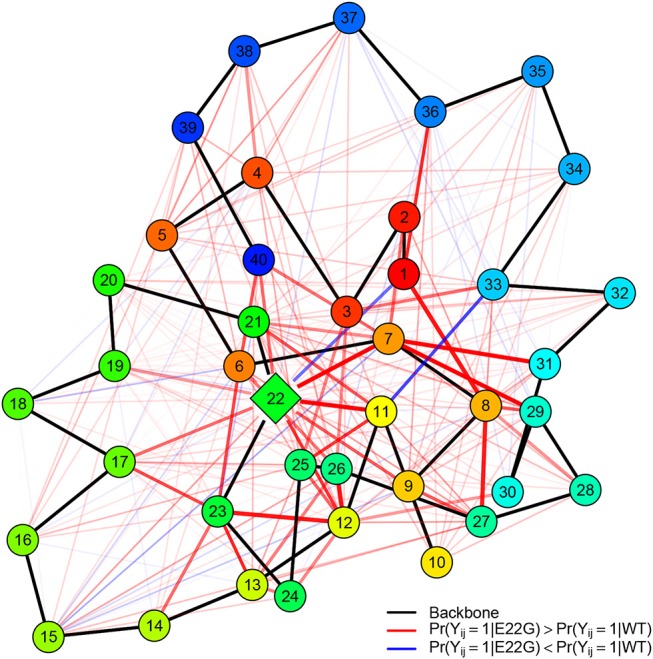
Relative PSN edge frequencies, E22G vs. WT. Of edges that differ statistically between E22G and WT, more of them are characteristic of the E22G variant (red) than wild type Aβ(blue); backbone edges (black) are provided as a reference, with vertices colored by residue index (red to blue). Edge width and translucency reflect the magnitude of relative difference in appearance rates (heavier edges differ more). Although E22G forms structure more readily in most locations, a key complex of activity involves residues 7, 8, 11, 22, and 23 (with several other residues also participating).

To get a better sense of how these differences in structure arise, it is useful to distinguish the residue contacts that arise more often in E22G than WT (and vice versa). Figure 10 shows, for both sets of PSNs, the edges that are found significantly more often in E22G (red) or in WT (blue). Mutation of the glutamic acid at position 22 to glycine clearly enhances a large complex of potential contacts, prominently including residues 7-8, 11-12, and 22-23 (among others); in addition, we see a weaker but more broad-based enhancement of contact rates throughout the protein, but particularly in the C-terminal region. By contrast, relatively few contacts are more prevalent in WT, among the exceptions being pairwise contacts between 1 and 22 and 3 and 11, as well as some relatively local contacts in the C-terminal region (appearing to involve interactions among nearby non-polar residues). Overall, the broad pattern suggests that in WT, E22 both blocks interactions among residues in its immediate vicinity and limits the ability of the two large patches of nonpolar residues within the C-terminal region to interact (with some of these instead participating more often in ephemeral internal interactions). In E22G, the replacement of the bulky glutamic acid with the small and highly flexible glycine appears to allow these previously blocked groups to interact with much higher frequency, raising the average local cohesion.

It should be noted that all of the above contacts are transient, with typical conformations being quite sparsely connected (though some do have considerable self-interaction). Thus, these patterns reveal biases or general tendencies in a fluctuating system, rather than the stable structures characterizing more typical proteins. This raises the question of which particular structures are more strongly favored for WT vs. E22G, to which we now turn.

#### 2.3.2. What Transient Structures Characterize the Difference Between WT and E22G?

The above give us some sense of where transient structure is being formed in WT and E22G, but they do not provide a strong holistic sense of which sorts of global structures are more characteristic of E22G vs. WT. For that purpose, we must consider the networks as a whole. To do this, we fit statistical models to the respective E22G and WT minima that identify the network features that are more or less enhanced for each variant. We do this by leveraging ERGMs (Hunter et al., 2008), parametric statistical models for graphs that allow direct representation of complex dependence among edges. Given a random graph *G*, defined on support 𝔾, we may write its probability mass function in ERGM form as

(1) $$\mathrm{Pr}(G=g|\theta ,t,X)=\frac{\exp(\theta^{T}t(g,X))h(g)}{\sum_{g^{\prime }\in G}\exp(\theta^{T}t(g^{\prime },X))h(g^{\prime })},$$

where *t* : 𝔾, *X* ↦ ℝ^*k*^ is a vector of sufficient statistics, θ ∈ ℝ^*k*^ is a parameter vector, *h* is a *reference measure* satisfying 0 ≤ *h*(*g*′) ≤ ∞ for *g*′ ∈ 𝔾 and *h*(*g*′) = 0 otherwise, and *X* is a set of covariates. In the case of residue-level PSNs, 𝔾 is the set of all simple graphs on *N* vertices (where *N* is the length of the primary sequence), subject to the constraint that each vertex is tied to the vertices corresponding to its neighbors in the protein backbone. Here, we follow typical practice for unvalued, fixed-*N* networks and take *h* to be the counting measure on 𝔾, implying that *h*(*g*′) ∝ 1 for *g*′ ∈ 𝔾 and 0 otherwise. Since *h* then cancels for graphs in the support, we henceforth omit it in our notation (it being tacitly assumed that the probability of graphs outside the support is 0).

An extensive statistical literature exists on ERGMs, and in particular on the problem of inferring an unknown θ from observations of *G*. Substantively, the model can be understood as describing *biases* in the distribution of *G* relative to the reference measure (in our case, the uniform distribution over possible 40-node PSNs), with the nature of each bias determined by the choice of statistics (*t*) and the direction and strength of each bias determined by θ. Here, we fit separate ERGMs to the sets of observed WT and E22G minima (respectively), inferring θ in each case by approximate Bayesian inference using Laplace parameter priors analogous to the L1 regularization employed in the well-known LASSO procedure (Tibshirani, 1996). Table 1 shows the posterior mean estimates, posterior standard deviations, and 95% central posterior intervals for the parameters (i.e., θ) of each fitted model. The estimated effects (i.e., *t*) are described in greater detail in section 4, but may be summarized as follows: an *Edges* effect sets the baseline PSN density; *Backbone Dist* indicates the effect of the absolute distance through the backbone (in units of residues) on the propensity of each residue pair to be in contact; *Hydophobicity* indicates the effect of hydrophobicity (as measured by the scale of Kyte and Doolittle, 1982) on the overall propensity of each residue to form contacts; *Charge Mixing* indicates the effect of like or unlike charges to be respectively in contact or not in contact (for charged residues); *Polar/Nonpolar Mixing* indicates the propensity of polar residues to be in contact with nonpolar residues; *Polar/Polar* mixing indicates the propensity of polar residues to be in contact with other polar residues; *Volume* indicates the effect of residue van der Waals volume (in Å^3^) on the propensity to form contacts; *Mass* indicates the effect of residue mass (in Da) on the propensity to form contacts; *Dist from Termini* indicates the effect of residue distance from the nearest terminus (ranging from 1 at the center to 0 at either terminus) on the propensity to form contacts; *GWESP(0.5)* indicates a geometrically weighted shared partner statistic with a decay parameter of 0.5, reflecting the tendency toward triadic clustering within the PSN; and *Prior Scale* refers to the scale of the Laplace parameter prior (which determines the strength of regularization).

**Table 1:** Posterior estimates for the WT and E22G PSN ERGMs (respectively).

	**Wild type**				**E22G**			
**Parameter**	**Post mean**	**Post SD**	**Q2.5%**	**Q97.5%**	**Post mean**	**Post SD**	**Q2.5%**	**Q97.5%**
Edges	−6.137	0.0719	−6.286	−5.986	−6.356	0.0667	−6.484	−6.218
Backbone dist	−0.025	0.0017	−0.028	−0.021	−0.019	0.0014	−0.021	−0.016
Hydrophobicity	−0.003	0.0038	−0.010	0.005	0.002	0.0039	−0.006	0.009
Charge mix	−0.999	0.0449	−1.083	−0.909	−0.996	0.0533	−1.108	−0.901
Polar/Nonpolar mix	−0.347	0.0320	−0.411	−0.285	−0.365	0.0295	−0.419	−0.308
Polar/Polar mix	−0.512	0.0531	−0.614	−0.410	−0.478	0.0465	−0.571	−0.393
Volume	0.004	0.0007	0.003	0.006	0.003	0.0007	0.001	0.004
Mass	−0.001	0.0007	−0.002	0.001	0.001	0.0007	0.000	0.002
Dist from termini	0.140	0.0239	0.097	0.188	0.190	0.0247	0.145	0.241
GWESP(0.5)	2.137	0.0235	2.090	2.182	2.205	0.0221	2.159	2.246
Prior scale	0.941	0.0102	0.922	0.960	0.958	0.0080	0.941	0.974

Of the estimated effects, all except for hydrophobicity and mass have 95% credible intervals that do not contain 0, and posterior means for both models are quite similar. Broadly, we may interpret the parameter estimates as follows. The low baseline density (as determined by the edges parameter) is compatible with the general observation that both WT and E22G are generally unstructured, with most residues having few non-backbone contacts at any given time. We observe a mild tendency for residues that are far from each other in the primary sequence to interact; the high flexibility of Aβ implies relatively little inhibition of long-range contacts, however, and the effect is fairly small. As would be expected on physical grounds, electrostatic and nonpolar effects are fairly large (with pairs of nonpolar residues relatively more likely to form contacts than pairs of polar residues or polar/nonpolar pairs). Volume also has a small effect on contact formation, with larger residues being somewhat more likely to have more contacts. Perhaps more interestingly, distance from the nearest terminus (equivalently, placement in the middle of the primary sequence) is a strong positive predictor of the tendency to form contacts, and there is a strong overall tendency toward clustering (as might be expected on geometric grounds). Thus, there is a net bias toward structure formation for the interior of the protein, despite its overall high mobility and lack of persistent secondary structure.

Although these models are highly simplified, they can be thought of as expressing approximate “force fields” describing the relative favorability of different PSN structures with respect to each variant. Drawing on this intuition, we may use the models to construct a log “favorability ratio” that, for a given PSN, measures the extent to which it is relatively favorable for E22G vs. WT. In particular, let ${\hat{\theta }}_{WT}$ be the estimated coefficients for the WT model, and ${\hat{\theta }}_{E22G}$ the corresponding coefficients for the E22G model. Then, for PSN *G*, the quantity

(2) $$\begin{array}{l} f_{WT}^{E22G}\left(G\right)={\hat{\theta }}_{E22G}t_{E22G}\left(G\right)-{\hat{\theta }}_{WT}t_{WT}\left(G\right) \end{array}$$

is the log favorability ratio for E22G vs. WT (where *t*_*E*22*G*_ and *t*_*WT*_ indicate the vectors of graph statistics for *G* evaluated for each respective sequence, the two having slightly different residue properties). It may be observed from Equation 1 that $f_{WT}^{E22G}\left(G\right)$ is equal to the log ratio of the probability of observing *G* under the two respective models, up to an additive constant that does not depend upon the PSN. Thus, while the absolute level of $f_{WT}^{E22G}\left(G\right)$ cannot be interpreted, differences in the log ratio for different choices of *G* are meaningful; in particular, if $f_{WT}^{E22G}\left(G\right)>f_{WT}^{E22G}\left(G^{\prime }\right)$, then PSN *G* is relatively favored by E22G vs. WT vis a vis *G*′.

**Figure 11:**
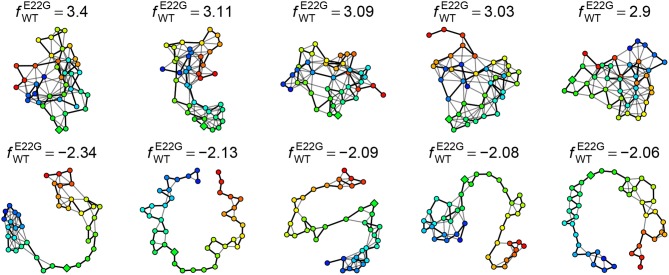
Five PSN structures most heavily favored by E22G **(Top)** and WT **(Bottom)**, respectively. E22G-favored structures are highly cohesive, while those favored by WT have little structure (most of which is local).

The log favorability ratio provides considerable insight into the types of transient structures that are most heavily favored by E22G relative to WT. For instance, Figure 11 shows the five PSN structures most favored by E22G and WT, respectively, out of all minima from both (pooled) sets. As can be seen, the minima most favored by E22G involve extensive, cohesive structures, while those favored by WT tend to be extremely sparse (with most structure being local with respect to the backbone). Interestingly, where the wild type-favored PSNs have more extensive structure, it tends to be near the termini (in contrast with E22G, which shows more extensive structure within the interior of the protein). As noted above, both models encourage structure formation within the interior of the primary sequence; however, wild type Aβ_1−40_ appears to favor conformations with terminal structure more than the E22G variant (plausibly because the E22G places far more probability mass on globally cohesive structures that are destabilized in the wild-type protein). Examination of these extreme cases thus gives us an immediate intuition for the nature of the subtle differences in transient structure formation that distinguish the two variants.

### 2.4. Comparative Cluster Analysis of WT and E22G Dynamics

Cluster analysis is a useful tool for subdividing conformational spaces, having been successfully employed in applications such as refinement of protein structure homology models (Raval et al., 2012), building Markov models for protein folding (Husic and Pande, 2017), and probing the configurational and hydrogen bonding structure of solvating water molecules in confined regions of proteins (Young et al., 2007). Here, we show how cluster solutions calibrated for accurate treatment of conformational dynamics combined with comparative analysis of cluster-induced transition networks can be used to reveal differences in the behavior of the WT and E22G Aβ variants.

#### 2.4.1. Can Differences in Physiological Temperature Dynamics for WT and E22G Be Detected?

It has been shown in the present study and elsewhere (Chebaro et al., 2015; Granata et al., 2015) that the thermodynamics of intrinsically disordered proteins are governed by vast potential energy surfaces with numerous or perhaps innumerable local minima corresponding to nearly isoenergetic microstates, rather than a single well-defined global minimum. This situation makes comparative analysis of thermodynamic distributions for similar IDPs extremely difficult compared to systems where only a few local minima exist. At the same time, experiments have confirmed that even subtly different IDPs, such as the WT and E22G proteins being studied in the present work, do exhibit a marked difference in their capacity to form amyloid fibrils (Lord et al., 2006; Norlin et al., 2012). This sharp contrast between the thermodynamic similarities of WT and E22G and the substantial difference in their behavior under solution conditions strongly suggests that there may be more easily discernible kinetic differences between them. In other words, although the configurations of both systems are distributed very similarly when time-marginalized, the way the proteins transition between regions of the conformation space may be distinct.

While the conventional intuition motivating clustering or segmentation of conformational space in the context of protein dynamics is that the protein will restricted to a relatively small number of low free energy basins (with relatively rare transitions over free energy barriers between basins) (Bolhuis et al., 2002), this cannot be assumed for IDPs: while local minima exist, they are extremely numerous and widely dispersed across a relatively flat energy landscape (Granata et al., 2015). However, even without the assumption of well-defined basins, we can segment conformational space into a set of discrete regions and use this as the basis for a coarse-grained treatment of protein dynamics (estimating transition rates from observed simulation trajectories). While many approaches could be used for this purpose, *k*-means clustering (Hartigan and Wong, 1979) on input space of torsion angles is a natural choice: it is highly scalable, adaptively places boundaries around regions of high conformational density, and leads to cells that are both convex and relatively compact. Here, we apply *k*-means clustering (using the R implementation R Core Team, 2018) to trajectories in torsion angle space produced by 500 ns long molecular dynamics simulations (10 × 10^6^ time steps each), jointly clustering WT and E22G to create a shared coarsening of their common conformational space. We then examine the dynamics on this coarsened space to reveal differences between the two systems.

#### 2.4.2. Choosing the Number of Clusters to Optimize Dynamic Accuracy

**Figure 12:**
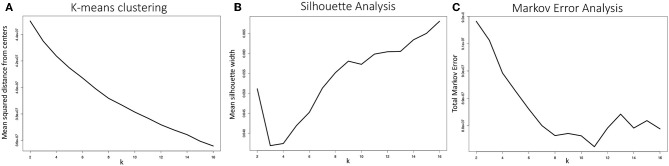
**(A)** Mean distance from cluster centers as a function of the number of clusters generated *k*, often called an elbow plot, is a typical metric for determining the ideal number of clusters to use in k-means clustering. In strongly clustered systems, the point where this mean distance drops abruptly, or the “elbow,” is the optimal value for *k*. Here we show the mean elbow plot for 20 separate clustering calculations using *k* values ranging from 2 through 16. **(B)** Mean silhouette width as a function of *k* is another common metric for choosing an optimal value of *k*. Although the metric shows that the optimal *k* value ought to be greater than 6, no clear optimum is demonstrated. The silhouette widths shown were generated by sampling 10 sets of 5,000 silhouette width values from all 20 clustering calculations and averaging. **(C)** Our metric for optimal *k* selection, whereby the quality of a given clustering was evaluated on the basis of how well a Markov model generated from a transition frequency matrix of transitions between clusters performed at predicting the actual trajectory's path through torsion angle configuration space. Here we see a clear optimum: *k* = 11, where the total Markov error metric is at a minimum.

An important parameter to determine in fitting any *k*-means clustering model is *k*, i.e., the number of clusters the algorithm will generate. One of the most common and straightforward metrics for determining the optimal choice of *k* is to plot the mean squared distance between the data points and their respective cluster centers, a.k.a. an *elbow plot*. For data sets with a strong characteristic number of clusters, a sharp decline in this distance will be observed when *k* is set to that characteristic number of clusters. As shown in Figure 12A, the configurations produced by the MD simulations of the WT and E22G variants of Aβ_1−40_ showed no well-defined elbow, a pattern compatible with a widely dispersed range of conformations with no deep potential energy wells. Although somewhat diminishing gains are observed somewhere between *k* = 5 and *k* = 10, this result is by no means conclusive, thus additional metrics for selecting *k* are needed.

Another commonly used metric for finding an optimal value of *k* for k-means clustering is to plot mean silhouette width as a function of *k* and look for a well-defined maximum (Rousseeuw, 1987). The silhouette width of a given data point *i* is defined as:

(3) $$\begin{array}{l} s\left(i\right)=\frac{b\left(i\right)-a\left(i\right)}{max\left\{a\left(i\right),b\left(i\right)\right\}} \end{array}$$

where *a*(*i*) is the mean distance between point *i* and all other points within its cluster, *b*(*i*) is the mean distance between point *i* and all points in the cluster it is nearest to but to which it does not belong. This equation produces silhouette width values −1 ≥ *s*(*i*) ≤ 1, where, on the extremes, 1 indicates ideal cluster membership for point *i* and -1 indicates that *i* has been grouped into the wrong cluster. Silhouette analysis of our system is shown in Figure 12B. Although the optimal choice of *k* is clearly greater than 8, again, the standard metric provides evidence for the wide dispersal of conformations, and a need to choose a *k*-selection approach that is tailored for the case of IDP trajectories.

Given that our goal is to segment a continuous conformation space for the purpose of building a coarse-grained approximation to the underlying dynamics, an alternative approach is to estimate the accuracy of the dynamic model produced by a given choice of *k*, and to find the *k* that leads to the lowest level of approximation error. Intuitively, the error involved in a Markov approximation of the true dynamics is dominated by two terms: the *coarsening loss* due to the approximation of each specific conformation within a voxel by the voxel centroid; and the *transition rate error* associated with imperfect estimation of the inter-voxel transition rates. Given a fixed set of trajectories, it is apparent that the coarsening loss is diminishing in *k*: the more finely we divide the space, the more accurately each observed conformation is represented. At the same time, however, larger choices of *k* also reduce the information available to estimate each inter-voxel transition rate, leading to errors that are increasing in *k*. Minimizing the total error is thus expected to lead to a *k* that optimizes the trade-off between coarsening and rate estimation errors.

To put these two error sources on an even footing, we unify them by defining a one-step *prediction error* for the coarsened Markov model. Specifically, given an observed conformation within a particular voxel, we predict the next conformation in the trajectory by (1) drawing the next voxel state from the Markov model, and (2) drawng a random conformation from the set of all observed conformations within the voxel. The distance between this drawn conformation and the observed next conformation is the one-step prediction error. Minimizing this error (summed over all observed transitions) automatically incorporates both the coarsening loss and the transition rate error, in a manner that is conceptually true to our end goal (approximating complex, high-dimensional conformational trajectories with a coarse-grained Markov model).

The one-step prediction error summed over all trajectories is referred to as the *total Markov error*, and is computed as follows. First, assume a set of observed trajectories, a clustering solution, and an estimated transition rate matrix. Next, begin with the first observed conformation, and proceed as follows:

1. Taking the current cluster ID as input to the Markov model, predict the cluster membership of the next time point.
2. Draw a configuration from the cluster into which the model predicted a transition.
3. Measure the distance between the predicted configuration and the actual configuration for that time step, and add that distance to the total Markov error.
4. Repeat steps 1 through 3 for the remainder of the trajectory, and either repeat with the next trajectory if any remain or else return the TME for that model.

The TME metric for *k*-means clustering was applied to 20 separate *k*-means model fitting calculations, varying *k* from 2 through 16 and averaged to produce the plot shown in Figure 12C. The metric shows a well-defined optimum at *k* = 11, where the total Markov error is at a minimum. The TME methodology implicitly strikes a balance in bias-variance tradeoff between the extremes of too few clusters, where transition frequencies are more likely to be well-sampled but the configuration draws from step 2 are drawn from higher variance clusters, and too many clusters, where smaller clusters have lower variance but under-sampling of transitions imparts a bias to the random walks in cluster space.

#### 2.4.3. Transition Frequency Graphs From k-Means Clustering of WT and E22G Trajectories

**Figure 13:**
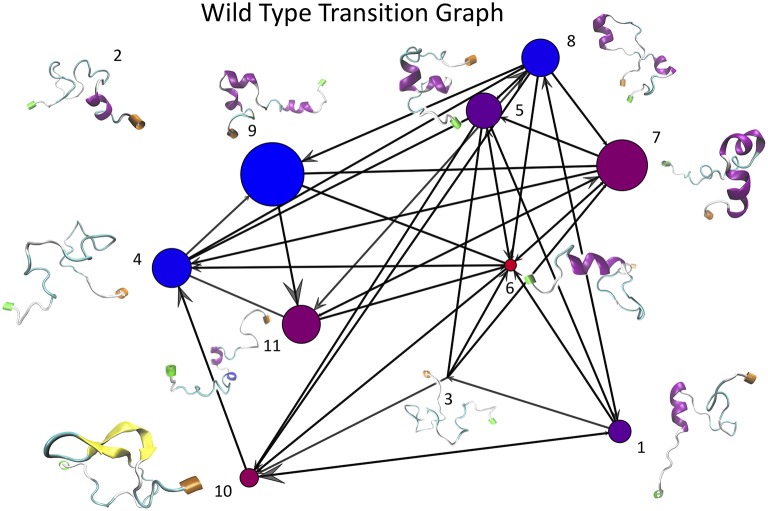
This graph of transition frequencies between clusters 1 through 11 was generated by clustering the trajectory in torsion angle space from a 500 ns simulation of wild type Aβ_1−40_. This particular clustering model was selected due to it having the minimum total Markov error (sum of both WT and E22G) of all 20 replicates having *k* = 11. Each vertex represents one cluster, edges indicate that transitions were observed between each respective pair of vertices, and molecular structures shown are the structures from the trajectories nearest each cluster center (N termini are labeled green and C termini are labeled orange). Note that this is a directed graph, thus the size of the arrowheads are proportional to the number of transitions that occurred in that direction. The size of each vertex is proportional to the number of configurations from the WT trajectory belonging to that cluster, and the color of each vertex represents the relative populations of WT vs. E22G in the aggregate of both trajectories (the bluer the more WT, the redder the more E22G).

**Figure 14:**
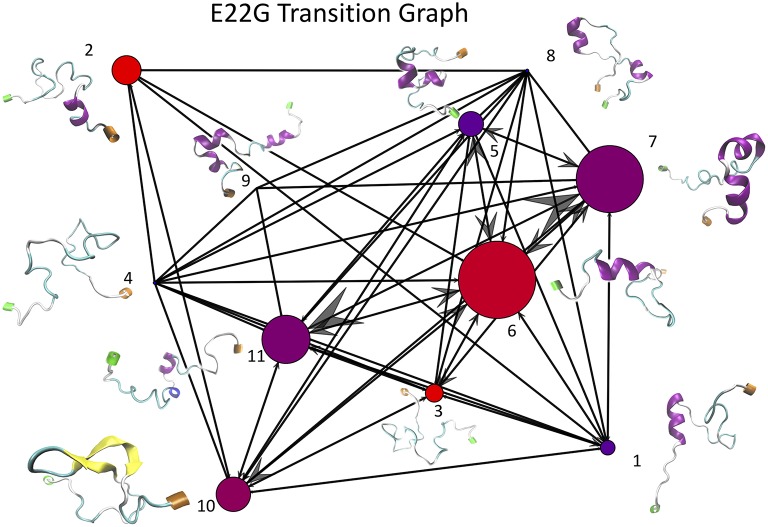
This graph of transition frequencies between clusters 1 through 11 was generated by clustering the trajectory in torsion angle space from a 500 ns simulation of E22G mutants of Aβ_1−40_. The clustering model used is the same as that shown in Figure 13, only applied to the E22G trajectory instead. The image convention used is also the same as that of Figure 13.

Once the optimal number of clusters of *k* = 11 was identified using the total Markov error metric, the lowest TME of the 20 *k*-means models with *k* = 11 was selected for further analysis. The matrices of transition frequencies between clusters (see section 4) are ideally represented using graphs (Figures 13, 14). A few key observations are immediately apparent when comparing Figure 13 with Figure 14. The E22G graph displays a much higher degree of connectivity compared to WT, with more evenly distributed populations across the clusters visited along its trajectory. Notably, cluster number 6, the highest populated cluster in the E22G transition graph, is both highly connected and minimally populated in the WT graph. This is noteworthy because although transitions were observed between cluster 6 and 9 of the 10 other clusters present in the WT trajectories, the trajectories did not remain in cluster 6 long enough to produce a more substantial population in that cluster. This implies that while cluster 6 is highly accessible to both WT and E22G variants, E22G appears to exhibit substantially higher stability in this region of configuration space.

**Figure 15:**
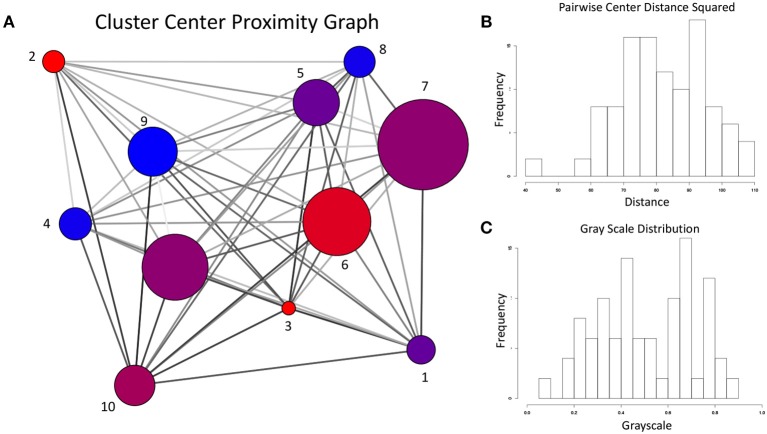
**(A)** For the cluster center proximity graph, the vertex color follows the same convention as Figures 13, 14, while vertex size is proportional to the aggregate population (both WT and E22G). The edges of this graph represent the proximity between their respective vertices in configuration space (the darker the edge, the closer the vertices are positioned in torsion angle space). **(B)** Distribution of the pairwise distances between cluster centers. **(C)** Distribution of grayscale values (0 is black, 1 is white) for the edges in **(A)** was produced by mapping the pairwise distances with a sigmoidal function. Note that the shape of the distribution is very close to that of **(B)**, indicating an accurate representation of the proximity data in the grayscale.

Given the sharp contrast between the transition frequency graphs in Figures 13, 14, it is necessary to examine the possibility that the difference in configuration space sampling is due to the trajectories being too short. More specifically, since the configuration space of Aβ_1−40_ is believed to be expansive, it is necessary to demonstrate that the observed differences are not occuring because the two variants simply did not have time to cover the distance between the configuration subspaces favored by one vs. the other. As a way to address this, we generated the cluster proximity graph shown in Figure 15. It is immediately obvious that this is a very well-connected graph, with many of the strongest ties occurring between vertices whose populations are dominated by differing variants. For example, note that most of the strongest ties in the graph are between nodes of substantially different relative populations of WT vs. E22G. As a specific case, consider the three most WT-dominant nodes on the graph, nodes 4, 8, and 9: all exhibit some strong ties, yet none of their respective strong ties are shared between each other. The cluster center proximity graph provides strong evidence that the disparity between the clusters sampled in the WT and E22G simulations are indeed inherent to their respective dynamics, and not an artifact of under-sampling.

## 3. Discussion

This comparative study of the wild type Aβ_1−40_ protein and its “Arctic” E22G variant identifies some key differences in the types of transient structures formed by monomers of the disease-related variant. Although the Ramachandran plots and angular velocity distributions of MD trajectories for these proteins are essentially identical, SVM analysis finds key sets of torsion angles that are indicative of conformations that are more characteristic of either wild-type or E22G. Combining this approach with PCA provides a more detailed view of the differences in transient structural motifs formed, namely the amount of helical character in the vicinity of residues 14-23 and the amount of contact between the C-terminal region and other parts of the protein. Comparisons of the similarity scores for the wild-type and E22G monomers investigated here with known fibril structures from the Protein Data Bank reveal that most of the known fibril structures occupy more wild-type like conformations, suggesting that E22G may fibrillize into a different topology, a hypothesis that is consistent with morphological differences in experimentally observed fibrils (Norlin et al., 2012), although high-resolution structures have not yet been solved for this variant. The previously discussed results approach the structures from torsion angle space, which is a convenient representation of backbone conformations, but does not address intramolecular connectivity. Protein structure networks (PSNs) enable a parsimonious representation of local and long-range cohesion. We find that the mean degree core number, a measure of each residue's connectivity, is significantly higher for most residues in E22G compared to wild-type, with particularly large differences observed in residues G15 to M35. This region of enhanced structural cohesion in the E22G variant may represent a nucleation site for the formation of pathological aggregates. PSN analysis of the five structures most favored by wild-type vs. E22G shows that the former prefers much sparser, extended structures, while the latter is prone to compact, densely connected conformations. Overall, this enhanced propensity of E22G to form denser patterns of inter-residue contacts, even if these species exist only transiently, is indicative of its increased susceptibility to aggregation. Our results not only provide insight into this protein system, but also illustrate a more general approach that can be applied to comparative analysis of intrinsically disordered proteins in other settings. While a strong precedent exists for applying frameworks devised for characterizing proteins with well-defined folded states, like DSSP (definition of Secondary Structure of Protein Joosten et al., 2010), toward characterizing the transient structure present in IDPs (Rosenman et al., 2013), we present a methodology that allows the latent structure of the data itself to define the metrics for similarity or difference between variants. Our approach does not risk the confirmation bias that can result from applying methods that search for a particular known type of order in an intrinsically disordered system. Rather, the ML-based methods shown herein search for the most predictive latent structure in the data and then maps that structure onto some intuitable paradigm. In most supervised machine learning applications, the goal is to train a classifier or regression model that can be used to make predictions on future data points after being trained on training data from the past. We have demonstrated that tools from the ML toolkit, such as SVMs and clustering algorithms, can be used in ways that go beyond traditional “black box” approaches, and instead be used to answer mechanistic questions about *how* and *why* subtle structural differences in complex systems like IDPs can lead to markedly different dynamics. Although fitting the models remains an important step in the present work, the utility of a well-trained ML model goes beyond being able to make accurate predictions. Using our approach, the fact that we are able to train a model to accurately classify or group structures as having WT or E22G character, given the training data, serves as an indication that the input data is indeed a set of sufficient statistics for discerning between the classes of interest. This is a key piece of information for molecular simulations in general, as one must always be wary that an inconclusive result is due to the inherent problems of molecular simulations, such as under-sampling or insufficiently detailed models. For example, in the case of the present work, wild-type Aβand the E22G variant are known to exhibit radically different fibrillization dynamics on experimentally accessible size and time scales, yet standard approaches to analysis of MD simulations of these systems show little to no difference in their behavior (e.g., the Ramachandran plot if Figure 1). As is the case for MD-based study, when standard methods of analysis are inconclusive, a legitimate concern is that lack of detail in the MD forcefields and/or under-sampling could be to blame for the inability to differentiate between WT and E22G dynamical data with the standard methods. By using multiple ML approaches to first prove that indeed enough simulation data is present to reliably differentiate between variants, and subsequently probe the ML models themselves to determine which input characteristics and even which specific configuration data points were most informative, we have demonstrated that our ML-based methods can be used to simultaneously verify the adequacy of the sampling while providing a less biased interpretation of the dynamics of intrinsically disordered proteins.

While there is no one-size-fits-all approach for characterizing the transient structure of IDPs–different questions demand different representations–we would suggest that several methods shown here are likely to prove widely useful in practice. As noted, we find residue-level PSNs to provide a fairly simple way to represent transient structure that complements traditional, secondary structure-based methods while capturing features that are hard to express via the latter. Measures of local cohesion (like the core numbers used here) are easily computed, and provide immediate insight into which regions of the protein tend to occupy locally folded conformations; comparing these measures across variants allows the impact of mutations on transient structure to be assessed without requiring formation of recognizable secondary structure. Model-based analysis of PSN structure using ERGMs is more complex, but provides a powerful tool for identifying transient structures that are differentially favored across variants. Given the rich analytic toolkit developed for the study of social networks (Wasserman and Faust, 1994; Brandes and Erlebach, 2005; Butts, 2008a) (which are themselves characterized by irregular and often transient structure), this would seem to be an area with substantial potential for further development.

## 4. Materials and Methods

### 4.1. Molecular Dynamics Simulation of AβMonomers

All MD simulations *Aβ*_1−40_ monomers were carried out using the NAMD 2.10 molecular dynamics software package (Phillips et al., 2005) with the CHARMM36 force field (Best et al., 2012) in Generalized Born implicit solvent (Qiu et al., 1997) with an electrostatic interaction cutoff of 14Å, an alpha (i.e., descreening) cutoff of 12Å, a 2fs step size, and an ionic concentration of 0.1M; except as noted below, all simulations were performed at constant temperature using a Langevin thermostat with a damping coefficient of 1/ps. The seed structure for WT *Aβ*_1−40_ was taken from the lowest energy conformation of the monomeric solution structure of (Paravastu et al., 2008) (PDB: 2LMN). The seed structure for the E22G variant was obtained via homology modeling using SWISS-MODEL (Schwede et al., 2003) (template PDB 2M4J Lu et al., 2013). Visualizations of the molecular structures were generated using the VMD software package (Humphrey et al., 1996), with additional processing performed using R (R Core Team, 2018).

#### 4.1.1. Identification of Local Minima

To obtain an overdispersed set of seed conformations, 100 ns MD simulations at 450K were carried out for WT and E22G, respectively using the above protocol; 1,000 conformations were collected in each case (1 per 100 ps), with the first being discarded and the rest being retained for subsequent analysis. Each conformation obtained from the above process was then used to seed a 1ns annealing trajectory in which temperature was systematically lowered from 310K to 0K by constant increments of 1K (i.e., with approximately 1,600 time steps between increments) using velocity reinitialization (no Langevin thermostat). The final conformation from each of 1998 annealing runs was retained as a local minimum for further analysis (resulting in 999 minima for each of WT and E22G, respectively).

#### 4.1.2. Simulation of Conformations and Angular Velocities from Dispersed Starting Points

To sample Aβ_1−40_ conformations across a wide range of conformation space, we use the above-identified local minima as seeds for short secondary trajectories at physiological temperature. For each minimum, we simulated 10 independent trajectories at 310K, using our base protocol. Each trajectory was simulated for 50 intervals of 2 ps, separated by “bursts” in which conformations were recorded 10 times separated by intervals of 20 fs. This resulted in a total length per trajectory of approximately 110 ps. In total, 9,990 trajectories were simulated for each of WT and E22G, with approximately 500,000 10-configuration “bursts” recorded for analysis. Mean angular velocities were then estimated for each burst by taking the mean of the circular (angular) difference between frames on each torsion angle and dividing by the interval between frames.

#### 4.1.3. Simulation of Dynamics at Physiological Temperature

To examine longer-range *Aβ*_1−40_ dynamics at physiological temperature, independent trajectories using our base protocol were simulated for WT and E22G at 310K for 500 ns. 250,000 conformations (1/2ps) were retained from each trajectory for subsequent analysis.

### 4.2. Support Vector Machine Analysis of Low-Energy Conformations

Backbone dihedral angles were obtained for all local minima using a combination of R and VMD scripts; for subsequent analysis, each torsion angle was represented via its real and imaginary components (for a total of 160 input features per conformation). SVM analysis was performed using the e1071 package for R (Meyer et al., 2018), using a Gaussian (aka radial basis function) kernel. Hyperparameter tuning for the kernel bandwidth and cost parameters was performed via a grid search using 10-fold cross-validation. For local analysis of mean angular differences across the decision surface, the set of all support vectors for the SVM solution was obtained and sorted into matched E22G/WT pairs by Euclidean distance in the input space (with the closest pair being matched first, then the next closest, and so on until no pairs remained). Angular (i.e., minimum circular) differences were then computed for the torsion angles in each pair, expressed as the angular displacement needed to go from the WT angle to its E22G counterpart (in radians).

For analysis involving fibrillar conformations, all models were extracted from PDB Berman et al. (2000) entries 2LMN (Paravastu et al., 2008), 2LMO (Paravastu et al., 2008), 2LMP (Paravastu et al., 2008), 2LMQ (Paravastu et al., 2008), 2LNQ (Qiang et al., 2012), 2M4J (Lu et al., 2013), 2MPZ (Sgourakis et al., 2015), 2MXU (Xiao et al., 2015), 5KK3 (Colvin et al., 2016), and 5OQV (Gremer et al., 2017). The conformation of each monomer in each fibril structure was extracted and converted to torsion angle features as described above. Because many reported structures were missing most or all of the N-terminal residues, we limited analysis to residues 15-40. A second SVM solution was obtained from the minima using only these residues using the above protocol, which was employed for this analysis. The projection of each fibril onto the feature space vector normal to the separating hyperplane (the “affinity score”) was performed by obtaining the decision value for the classification prediction (E22G vs. WT) for each fibrillar conformation. To obtain information on the mean gradient of the affinity score over the fibrillar conformations, scores were regressed on the input features of the conformations; the resulting coefficients estimate the mean gradient of the affinity score for the real and imaginary portions (respectively) of each torsion angle, averaged across conformations. For visualization, the two coefficients for each torsion angle were transformed into modulus/argument representation [i.e., for torsion angle θ_*i*_, $\beta_{i}sin\left(\theta_{i}\right)+\beta_{i}^{\prime }cos\left(\theta_{i}\right)=b_{i}sin\left(\theta_{i}-y_{i}\right)$ with $b_{i}=\sqrt{{\beta_{i}}^{2}+{\beta_{i}^{\prime }}^{2}}$ and $y_{i}=\tan^{-1}\left(\beta_{i}^{\prime }/\beta_{i}\right)$]. All calculations were performed using R (R Core Team, 2018).

### 4.3. Protein Structure Network Analysis

Residue-level PSNs were obtained for each local minimum conformation by calculating distances among all atom pairs and forming an edge between residues *r*_*i*_ and *r*_*j*_ if there existed atoms *a*_*i*_ ∈ *r*_*i*_, *a*_*j*_ ∈ *r*_*j*_ such that the *a*_*i*_, *a*_*j*_ distance was smaller than 1.2 times the sum of their van der Waals radii. All analysis and visualization was performed using R and statnet (Handcock et al., 2008; R Core Team, 2018); van der Waals radii were taken from Alvarez (2013). *k*-cores were calculated for all PSNs using the sna library for R (Butts, 2008b).

ERGM estimation was performed using an approximate Bayesian procedure building on the approach of Desmarais and Cranmer (2012). We independently estimate a model for each sample of PSNs, with the structure

$$\begin{array}{l} \sigma \sim \text{Inv}-\text{Gamma}\left(\kappa ,\zeta \right)\\ \theta_{1},\ldots ,\theta_{p}\sim \text{Laplace}\left(0,\sigma \right)\\ Y_{1},\ldots ,Y_{n}\sim \text{ERGM}\left(\theta ,X\right), \end{array}$$

where σ is the prior scale (with hyperparameters κ and ζ), θ = (θ_1_, …, θ_*p*_) is the vector of ERGM coefficients, *Y* = (*Y*_1_, …, *Y*_*n*_) is a PSN sample, and *X* is a set of protein-specific covariates (e.g., residue properties). Draws at each level are taken to be conditionally independent. Intuitively, this model is a Bayesian analog to the LASSO procedure applied to a pooled ERGM, with the Laplace parameter priors inducing the equivalent of L1 regularization on the posterior mode. (To improve regularization performance, we rescale the changescores associated with θ to unit variance during the estimation process, so that each coefficient is on the same scale; reported estimates have been returned to the original scale). Because direct posterior simulation for this model would be prohibitively computationally expensive on the large sample of networks used here, we instead employ an approximate inference strategy closely related to that of Schmid and Desmarais (2017) for single networks and Desmarais and Cranmer (2012) dynamic networks. Our approach proceeds as follows. For a specific sample, *Y*, we approximate the posterior mode θ|*Y, X* by numerically maximizing the quantity

$$\int_{0}^{\infty }p(\theta |\sigma )p(\sigma |\kappa ,\zeta )\prod_{i=1}^{n}PL(Y_{i}|\theta ,X)d\sigma$$

where PL is the conditional *pseudo-likelihood* of *Y*_*i*_ (Strauss and Ikeda, 1990) given the constraint that all residues must be adjacent to their neighbors along the protein backbone. The pseudo-likelihood is an easily calculated approximation to the exact ERGM likelihood whose mode, for large conditionally independent samples, approaches that of the true likelihood (Strauss and Ikeda, 1990). To obtain approximate posterior quantities, we then perform Bayesian bootstrap (Rubin, 1981) simulation of θ|*Y*^(*j*)^, *X* over replicates *Y*^(1)^, …, *Y*^(*m*)^ of the original data set (with graphs as the independently resampled units). We report approximate posterior mean, standard deviations, and 95% credible intervals obtained through this procedure for θ and σ.

Model terms used for the PSN ERGM analysis were computed using a combination of R scripts and tools within the ergm statnet package (Hunter et al., 2008); descriptions for model terms used here follow e.g., Morris et al. (2008). A standard *edges* term was used as a density offset, with an *absdiff* term for distance along the backbone, and a *nodemix* for polar/nonpolar interaction (with nonpolar/nonpolar as the reference category). Electrostatics were implemented via an *edgecov* term with a covariate matrix *Z* such that *Z*_*ij*_ = 1 if *r*_*i*_ and *r*_*j*_ have the same nonzero charge, *Z*_*ij*_ = −1 if *r*_*i*_ and *r*_*j*_ have the different nonzero charge, and *Z*_*ij*_ = 0 if either *r*_*i*_ or *r*_*j*_ are uncharged. *nodecov* terms were included for hydrophobicity (using the scale of Kyte and Doolittle, 1982), residue volume (in Å^3^), residue mass (in Da), and residue-wise distance from the nearest terminus (scaled from 0 to 1). Finally, we account for endogenous clustering using a fixed-decay geometrically weighted edgewise shared partner term (*GWESP(0.5)*). For the Laplace scale, we employ a minimally informative (i.e., diffuse) hyperprior (κ = 0.1, ζ = 1.1).

Computation for the log relative favorability ratio was performed for each PSN by calculating the model statistics (i.e., terms) for the adjacency structure of the PSN under the respective residue properties of each variant and then multiplying by their respective parameter estimates per equation 2. $f_{WT}^{E22G}$ was then calculated for all WT and E22G minima PSNs, with the highest and lowest scoring configurations (respectively) being chosen for visualization.

### 4.4. Comparative Cluster Analysis of WT and E22G Dynamics

All *k*-means clustering was carried out using the standard R implementation of *k*-means clustering (R Core Team, 2018). Torsion angle vectors used to define the configuration space were expanded into real and imaginary components, as outlined in section 4.2. The Markov models for the total Markov error metric were generated matrices of transition frequencies by defining a Jeffreys prior on each row, with the observed transitions for that row treated as multinomial data, leading to a posterior mean for the *c*_*ij*_ transition of (*Z*_*ij*_ + 0.5)/(*N*_*i*_ + *k*/2), where *N*_*i*_ is the number of cluster pairs starting in *c*_*i*_ and *Z*_*ij*_ is the total number of transitions from cluster i to cluster *j*.

## Data Availability

The raw data supporting the conclusions of this manuscript will be made available by the authors, without undue reservation, to any qualified researcher.

## Author Contributions

Simulation, analysis, and method development were performed by GG and CB. GG, CB, and RM wrote the paper.

### Conflict of Interest Statement

The authors declare that the research was conducted in the absence of any commercial or financial relationships that could be construed as a potential conflict of interest.
